# British Muslims' perceptions of therapy with non‐Muslim therapists: A qualitative analysis of survey responses

**DOI:** 10.1111/papt.70023

**Published:** 2025-11-14

**Authors:** Hibah Hassan, Sarah Lack, Paul M. Salkovskis, Graham R. Thew

**Affiliations:** ^1^ The Oxford Institute of Clinical Psychology Training and Research University of Oxford Oxford UK; ^2^ Oxford Health NHS Foundation Trust Oxford UK; ^3^ Department of Experimental Psychology University of Oxford Oxford UK

**Keywords:** barriers, CBT, Muslim, qualitative, therapy, United Kingdom

## Abstract

**Objectives:**

Accessing psychological therapy presents unique challenges for Muslims, who are underrepresented in primary care mental health services in the United Kingdom. This qualitative study sought the narratives of British Muslims to gain insight into the perceived barriers and facilitators to engagement with therapy.

**Methods:**

Eighty participants responded to an online free‐text survey question enquiring about their views on therapy with a non‐Muslim therapist after completing a short experimental study. A structured tabular approach grounded in reflexive thematic analysis was used to analyse data.

**Results:**

Three main themes were identified in our analysis: ‘*Fundamental differences*’, ‘*It's not worth the risk*’ and ‘*Overcoming barriers*’. Within these themes we explore perceived interpersonal and systemic tensions, as well as facilitators to engagement with psychological therapy.

**Conclusions:**

These findings are discussed in terms of implications for clinicians working with Muslim clients, with a focus on the importance of understanding and attending to faith and relational concerns.

## INTRODUCTION

Integrating and attending to clients' values in therapy is understood as key to strong therapeutic alliances and successful outcomes. In the face of differences between therapist and client, this may be even more important. For example, there is a growing recognition of the need to integrate and attend to faith and religion when working with clients for whom these are important, especially when therapists themselves may not be religious (Hassan et al., 2024; Pargament & Lomax, 2013; Post & Wade, 2009).

There is some evidence to suggest that religious clients hold expectations that their therapists should know how to discuss religious issues and believe that good therapists are sensitive to their religious beliefs (Oxhandler et al., 2021). Other studies have found that religious clients express a desire for holistic treatment in which religion is not only considered but also *incorporated into* treatment (Ayub & Macaulay, 2023; Harris et al., 2016; Weatherhead & Daiches, 2010).

Despite this, although some mental health professionals may recognise religion as integral to the work of therapy, discussing religion can be uncomfortable for some therapists, with some feeling unable to have these important conversations altogether (Crossley & Salter, 2005; Knox et al., 2005; Mayers et al., 2007; Rose et al., 2001).

This possible disparity between client and therapist preference may explain why some religious clients express negative attitudes about engaging with therapy services. Clients express concerns that therapists may either ignore their religion, or approach their religious beliefs insensitively, or fear that they may be exposed to conflicting or anti‐religious beliefs (Mayers et al., 2007). This may lead to a mutual avoidance of acknowledging and incorporating religious beliefs into formulation and treatment, and leave clients with the view that their therapists are limited in their ability to help (Mitchell & Baker, 2000) or that services are irrelevant to their needs (Weatherhead & Daiches, 2010). This may in part explain why religious clients are underrepresented in secular mental health services, as is evident in the United Kingdom (Office for National Statistics, 2022).

One proposed solution is client‐therapist ‘matching’, whereby clients and therapists are matched on factors including religiosity. Although matching appears to be a preference for a number of religious clients (Cragun & Friedlander, 2012; Dimmick et al., 2020; Dimmick et al., 2022), it does not always improve client outcomes, as seen in a study on Orthodox Jewish patients (Rosmarin & Pirutinsky, 2020). Moreover, other studies find that client preferences for the way in which client and therapist are matched may vary. For example, in Gregory et al.'s (2008) vignette study, participants (especially those who rated themselves high in religiosity) reported a preference for psychologists who were affiliated with any major religion over atheist psychologists, and in a study by Mayers et al. (2007 cited in Post & Wade, 2009), religious clients held mixed views on whether therapists should be matched to their religious beliefs, but found the therapeutic alliance strongest when experiencing therapists accepting and respecting their beliefs.

These findings underscore the need to better understand the attitudes and perceptions religious clients have of therapy. In the United Kingdom, this is particularly necessary, where it has been shown that individuals from religious backgrounds are marginalised in their access to treatment for mental health problems (Office for National Statistics, 2022). In particular, British Muslims are underrepresented in primary care psychological services (NHS Digital, 2020), have less positive attitudes toward seeking professional psychological help (Hamid & Furnham, 2013), and have poorer treatment outcomes than service users from other religious backgrounds (Baker & Kirk‐Wade, 2024; Mir et al., 2019).

The limited literature on how British Muslims perceive therapy within secular services indicates that it is timely and necessary to explore the barriers to accessing talking therapy for this group. In their interview study of 14 British Muslims, Weatherhead and Daiches (2010) discuss themes on the importance of therapy content, therapist characteristics and the relevance of mental health services that were identified when clients had strong faith. Similarly, participants in Ayub and Macaulay's (2023) study expressed a wish for religion to be integrated into mental health services. Broader sociopolitical barriers may be relevant for this group; Byrne et al. (2017) employ the ‘Circles of Fear’ formulation developed by Keating et al. (2002) to argue that the ‘othering’ of Muslims in British society may have contributed to the erosion of trust between Muslim communities and health and education systems.

The current study set out to explore how British Muslims, comprised of several different ethnic groups, perceive therapy with a non‐Muslim therapist.

## METHODS

### Design and Procedure

This study reports the qualitative findings of a larger project aiming to understand the barriers and facilitators to therapy for British Muslims. Participants were asked for their ‘views on therapy with a non‐Muslim therapist’ via an online text box, which was the final component of an experimental study designed to evaluate the impact of acknowledging religion as a value in Cognitive Behavioural Therapy (CBT). Participants were given information about CBT via a video vignette and were subsequently shown a video in which the same therapist either mentioned the importance of acknowledging clients' general values in therapy, or explicitly mentioned acknowledging religion as part of therapy (see Hassan et al., 2024 for more information). In that study, scores on perceived credibility of therapy and treatment expectations were significantly higher when religion was explicitly mentioned by the ‘therapist’, but acknowledging religion did not impact anticipated alliance. The present study analyses the free responses of those participating using Thematic Analysis.

The study was approved by the University of Oxford's Central University Research Ethics Committee (MRA‐19/20‐19,878).

### Participants

Participants were adult British Muslims over the age of 18 who had no current or past experience of receiving or delivering psychological therapy. Participants were recruited online between June and August 2022 through advertisements on social media, including Twitter, LinkedIn and WhatsApp.

Participants who clicked on a link for the study were taken to a consent page. Only participants who consented could proceed with the study. All responses were anonymous.

A total of 136 participants responded to the text‐box question asking: ‘Do you have any comments on your views of therapy with a non‐Muslim therapist (including any comments about the videos or your experience of completing this study)’. Of these, 40 responses were excluded due to responses such as ‘no comment’ or ‘N/A’. A further 16 were excluded due to comments related to the experience of completing the study, such as technical issues or suggestions for improvements to the study design. Therefore, 80 responses were analysed.

Table 1 shows respondent demographics. The majority of respondents were female, Sunni and aged between 18 and 50. Most respondents were Asian or Asian British, including respondents from Pakistani, Bangladeshi, Indian, Kashmiri and Malaysian backgrounds, 8 respondents were African, African Asian or Black British, including Ghanian, Somali, Algerian and Kenyan, 8 respondents were Arab or Arab‐Mixed heritage including Iraqi and Arab‐other, 4 identified as White British, 3 participants identified as ‘Other’, including Kurdish and Turkish.

**TABLE 1:** Participant Demographics.

Demographic variable	*N*	%
Age		
18–20	14	17.5
21–30	26	32.5
31–40	20	25
41–50	16	20
51–60	2	2.5
61–70	1	1.25
Religious Sect		
Sunni	72	90
Shia	4	5
None/prefer not to say	4	5
Ethnic group		
Asian or Asian British	57	71.25
African, African Asian or Black British	8	10
Arab or Arab Mixed	8	10
White British	4	5
Other	3	3.75
Gender		
Female	52	65
Male	27	33.75
Prefer not to say	1	1.25

### Analysis

The standardised steps of reflexive thematic analysis (Braun & Clarke, 2006, 2019) were used to analyse brief text data produced by this study, including familiarisation with the data, generating initial codes, developing themes, reviewing themes, defining and naming themes and writing the report. To effectively explore a range of possible themes and patterns within a large dataset, guidance from Robinson's (2022) structured tabular approach to conducting thematic analysis was used. This methodology involves using Microsoft Excel to visualise and organise large amounts of brief text data to follow the process of thematic analysis.

In the experimental study (see Hassan et al., 2024), participants were shown a video in which the same therapist either mentioned religion explicitly as part of therapy, or referred to attending to patients' general values. Subsequently participants' qualitative data were separated into two groups: those who had seen the video in which religion was acknowledged, and those who had seen the video in which religion was not acknowledged. Both data sets were exported into Microsoft Excel and potentially identifying information was removed. Deep immersion in the data was achieved through repeated reading and re‐reading of participants' responses with initial notes also taken in this phase. Initial codes were then identified following an iterative process of reviewing, coding, reviewing and amending codes. Data under each code were collated and compared for similarities and discrepancies. From these initial steps, it was clear that the narratives across both video groups contained shared experiences, views and perspectives. As all participants were asked the same question and the codes and preliminary themes across the data sets captured shared views, the two data sets were combined.

The initial codes were reviewed again, and codes with perceived connections were then organised into preliminary themes. Attention was paid to ‘negative cases’ or data which might challenge the themes. Themes were then named to demonstrate which aspect of the data they described and how they related to the study question. Participants have not been allocated numbers or pseudonyms, as no participant is quoted more than once.

A critical realist position was adopted: we assumed that the participants' motivations, perspectives and experiences reflect a truth about their world, while recognising that these are filtered through the researcher's own assumptions and experiences.

The main researcher is a British‐Pakistani Muslim woman. This placed her as an ‘insider’ to the participants as she holds in mind her own personal experiences of family members and friends engaging or not engaging with therapy services. She also considered her own personal opinions on therapy with a non‐Muslim therapist and paid particular attention to when participants either shared similar or opposing views to her own. Her position was considered throughout the analysis. Bracketing exercises, including reflective discussions with colleagues, were conducted to raise awareness of potential biases and blind spots.

## FINDINGS

Across the range of views expressed by British Muslims in this study, we identified three main themes. In Theme 1, we explored perceived interpersonal differences between Muslim clients and non‐Muslim therapists that get in the way of building meaningful alliances. For some participants, these differences related to relational fears and systemic mistrust, captured in Theme 2. Some participants shared ways in which these differences and fears could be overcome; these facilitators are summarised in Theme 3.

### Theme 1: Fundamental Differences

Participants identified ‘*fundamental beliefs that are different*’ between themselves and non‐Muslim therapists, including differences in ‘*world view*’, and ‘*values and purpose in life*’. Consequently, some participants described feeling ‘*more comfortable’* with a Muslim therapist who they believe would understand them better, as this participant explains:

I as a practicing Muslim would prefer to see someone from within my own faith, as they may have a better understanding of my position as we are situated within the same framework

This view was shared by other participants who described it as ‘*much easier*’ to work with a Muslim therapist. Here, this participant explains how differences between themselves and a non‐Muslim therapist exist at the core of their motivations:

I don't think they can be fully invested in me if they don't understand and share my motivations for self‐purification ‐ a non‐Muslim therapist might help me fix the problem at the surface level but I'm not confident they will be able to get to the root of the problem

In referencing the therapist's lack of full ‘investment’, this participant appears to make assumptions that a non‐Muslim therapist's connection with them may be compromised or superficial. Similarly to this participant, a number of participants located the ‘root’ of mental health problems in a faith‐based understanding: this conceptualisation of mental health and faith as interconnected was one that participants felt a non‐Muslim therapist would not be able to attend to. This is exemplified here by this participant aiming for the ‘Islamic end goal’ of attaining God's pleasure:

Unless she (the therapist) herself believes in the Islamic end goal, she cannot truly help you on a journey she does not see the target to. Some companions are able to help partially yes, but the true companion is the one who walks with you to the destination IMO. [in my opinion]

The differences in belief system of therapist and participant means the therapist here is unable to be a ‘true companion’ so can only ‘partially’ help. This idea of was shared by other participants, as one expressed ‘*there will always be a degree of a block between myself and a non‐Muslim therapist*’.

Some participants extended the differences between themselves and a non‐Muslim therapist to differences in beliefs about how mental health should be addressed. Some participants described faith‐based mechanisms to cope with distress:

We already have strategies for coping with stressors in the form of trust in Allah (God), salah [obligatory prayers], dua [calling out to God], strong family and community ties etc.

In suggesting that ‘*we already have strategies*’, this participant suggests that therapy is not felt to be needed in the face of their faith, family and community support. Others likened connecting with God to a form of therapy that is built into the faith already:

As Muslims, we have been taught a great importance of connecting with Allah, and so created a kind of therapy where we share about feelings and wishes in Duas…

In identifying their own ‘kind of therapy’ this participant draws attention to the role of connecting with God in addressing mental health. This kind of therapy is felt to be different to that provided by secular services. Crucially, the differences identified in this theme were felt to impact participants' perceived ability to connect with or be understood by a non‐Muslim therapist.

### Theme 2: It's Not Worth the Risk

For some participants, perceived differences between themselves and a non‐Muslim therapist had implications for not only their ability to build meaningful relationships but also for their sense of safety in therapy. Risks including being judged, experiencing prejudice or discrimination, being given advice that is contrary to faith beliefs, as well as being reported to authorities are exemplified in this theme.

Participants shared worries about how a non‐Muslim therapist may perceive them and their faith, as this participant describes:

Non‐Muslim people tend to just not understand what it really means to be religious, and without realising it they tend to harbour quite patronising attitudes towards religious people

This participant draws attention to perceived unconscious biases that may be held by a non‐Muslim therapist, resulting in expecting a therapist to have ‘*patronising attitudes’* when discussing religion. In describing these attitudes as being ‘harboured’, this participant paints a picture of negative beliefs that are held covertly and that trust may be difficult to ascertain.

This fear of judgment was shared by others, as this participant expresses:

I would be worried that a non‐Muslim therapist would be judgemental about my faith, I'm a very conservative Muslim and believe that sharia [Islamic law] should be adhered to strictly

Other participants went further and shared worries about the risk to their faith if they worked with a non‐Muslim therapist:

(I) May feel uncomfortable when discussing Islamic issues with a non‐Muslim as they may give me advice that may go against my religion

Here, the therapist is framed as giving advice from a position of misunderstanding; the advice therefore may be unapplicable, or at worst, be harmful to their faith.

Two participants in this study referenced the threat of being reported to the authorities. This participant describes the threat of being reported to *Prevent*, the UK Government's national counter‐terrorism programme:

…(I) theoretically may think it [therapy] is useful, but worry about being referred to Prevent etc. Just for being a Muslim and for Muslim cultural or religious practices…this would affect my ability to safely access therapy as it's not worth the risk

For this participant, the risk of being reported to the authorities overshadows any potential benefits of therapy. They highlight the risk of being reported *‘Just for being Muslim’* suggesting that this alone is enough to raise suspicions.

Some participants explicitly named a sense of mistrust toward non‐Muslim therapists, stating: ‘*I would probably never be completely open with them [non‐Muslim therapist]*’, ‘*people feel safer with a fellow Muslim’, ‘there is a trust issue to work with non‐Muslim therapists’*. Issues of trust and safety identified in this theme pertain to wider systemic issues of Islamophobia, governmental policy and a mistrust of health care services' ability to accept religious differences.

### Theme 3: Overcoming Barriers

In addition to barriers, participants also shared factors which would facilitate engagement with services and positive therapeutic outcomes. For most participants, this was a sense that the therapist understood, respected and was able to incorporate faith into treatment. The extent of this varied between participants; some wrote of the value of a ‘*spiritually connected therapist*’, and others of ‘*a non‐Muslim who has some knowledge of Islam*’. Ultimately, the incorporation of religion was felt to ‘*overall increase the chances of success*’. Here a participant explains the importance of their therapist understanding faith and culture to build a strong connection:

If I were to consider engaging in therapy, connecting with the therapist is very important to me. The therapist doesn't necessarily need to be Muslim but having a good understanding of faith and culture will help build a good working relationship

Alternatively, here a participant describes their desire for therapy to reflect Islamic principles and values:

…it makes me as a practising Muslim far more comfortable if the therapist respects my preference for an Islamic application of CBT, so that it can reflect the principles and values that are important to Muslims…

This participant qualifies their preference for an ‘Islamic application’ of CBT as one which reflects the principles and values that are important to them. Other participants identified some advantages of having a non‐Muslim therapist, but with the precondition that their beliefs and principles were incorporated into therapy:

I actually think that getting a varied viewpoint is helpful, so having a non‐Muslim therapist may be helpful if they are truly understanding and incorporating Islamic beliefs and principles

Some participants shared other characteristics that were felt to be important to them in a therapist, including cultural sensitivity, gender matching and lack of judgement and preconception. The therapists' level of skill and professional qualities were also important, and for a small minority of participants, seen as more important than their identification with faith.

I think the religion of the therapist is not massively relevant, what's most important is the ability to understand deeply how someone is feeling and be empathetic with a good set of tailored support

## DISCUSSION

This study aimed to provide an initial synthesis of how British Muslims perceive therapy with non‐Muslim therapists following participation in an experimental design in which Cognitive Behavioural Therapy was described. Despite finding in the quantitative component of this study that British Muslims viewed therapy more favourably when religion is acknowledged (Hassan et al., 2024), central to our analysis of the qualitative data is an overarching tension between faith and therapy services: this is not to say that participants felt the process of therapy was incompatible with their faith, but rather that they may be misunderstood, unable to connect with, and possibly at risk of harm from a non‐Muslim therapist. Situated in the context of relational mistrust across sociopolitical spheres, the findings from this study suggest that British Muslims may experience feeling marginalised in their access to therapy, a felt sense of ‘otherness’, and perceptions that services cannot attend to their need of incorporating religion into treatment.

The desire for a therapist who ‘understands deeply’ is a thread that is woven throughout our analysis. Participants in our study expressed a variety of views about what it means to be understood or to be connected with, from wanting the therapist to share their fundamental beliefs about the purpose of life, to feeling that the therapist understood them deeply despite differences in religious beliefs. Given the diversity in our sample, our findings speak to a range of wishes and expectations, but central to these seems to be a desire to connect to a therapist who is able to consider and where appropriate, incorporate important belief systems into treatment.

Our findings align in part with suggestions by Rosmarin and Pirutinsky (2020) that for the provision of religiously sensitive treatment, a religion‐affirmative or favourable attitude toward religion is more important than the specific religious identity of the therapist. A religion‐affirmative approach may also reassure clients worried about being discriminated against as noted also in Inspirited Minds (2024). These findings from the present study also add to the existing literature on how British Muslims perceive therapy such as Weatherhead and Daiches (2010) who found that participants emphasised the value of faith being respected and acknowledged in therapy, and participants in Ayub and Macaulay's (2023) study who expressed a desire for services that not only consider spirituality but integrate it into treatment.

Our analysis also draws attention to systemic relational barriers that exist between British Muslims and therapy services. Similar findings are also illustrated in Muir's (2016, cited in Byrne et al., 2017) qualitative study on barriers to talking therapies in British Bangladeshi and Somali communities which found key themes of ‘landscapes of mistrust’, as well as Hammad et al. (2020) which found a mistrust of all statutory services in a group of British Muslims in London. In our study, two British Muslims explicitly referenced the threat of being reported to Prevent, a fear not unfounded given that British Muslims are profiled, and publicly stigmatised as ‘suspect’ (The People's Review of Prevent, 2022). Younis and Jadhav (2019) argue that healthcare settings reflect fears held in the public consciousness, so British Muslims accessing therapy may view therapists as suspicious of them. We support suggestions by Byrne et al. (2017) that therapists must be conscious of, and if needed, engage explicitly with the political context in which their clients are situated; non‐Muslim therapists must sensitively explore fears their Muslim clients may hold about engaging in therapy with non‐Muslim therapists, and focus on building trust from this perspective.

The main clinical implication of this study is that therapists must not avoid the topic of religion if it is important to their client. Bringing religion into the therapy room includes showing a willingness and taking responsibility for engaging clients in these conversations, naming differences between therapist and client worldviews, anticipating potential ruptures and repairs, including religion in formulation and goals for treatment, and holding in mind the continuum of preferences that have been highlighted in this study. Therapists may benefit from training on how to broach these topics, and how to use differences in faith and culture to foster strong alliances. At a service level, it may be beneficial to review how faith is considered at each stage of treatment pathways, and how the service is advertised in leaflets, posters and websites.

This study is limited by the use of one broad question following an experimental design, which meant that we were unable to probe for further information or contextualise this data. It is notable, however, that this study still produced large amounts of rich data, with a number of participants responding with lengthy paragraphs in response to the question. The sample was predominantly made up of participants under the age of 50 and may therefore not capture differences in generational perspectives and cohort beliefs.

This study is also limited to perceptions of therapy following a short video vignette of a therapist describing CBT instead of the lived experience of engaging in therapy, and the present sample of participants was not actively seeking therapy. Future research may wish to explore in greater depth the experiences of therapy British Muslims have had with non‐Muslim therapists to ascertain what meaningful integration of religion into therapy looks like for clients. Intergenerational differences and the impact of the level of education may be important contextualising factors. Furthermore, qualitative studies on the experiences of therapists working with Muslim clients would also be valuable in understanding the barriers faced by professionals.

## AUTHOR CONTRIBUTIONS

**Hibah Hassan:** Conceptualization; methodology; data curation; formal analysis; writing – original draft; writing – review and editing; project administration. **Sarah Lack:** Writing – review and editing; supervision. **Paul M. Salkovskis:** Supervision; writing – review and editing. **Graham R. Thew:** Conceptualization; formal analysis; supervision; writing – review and editing; writing – original draft.

## FUNDING INFORMATION

This project is supported by the National Institute for Health and Care Research (NIHR) Oxford Health Biomedical Research Centre [NIHR203316]. The views expressed are those of the authors and not necessarily those of the NIHR or the Department of Health and Social Care.

## CONFLICT OF INTEREST STATEMENT

The authors declare no potential conflicts of interest with respect to the research, authorship, and/or publication of this article.

## Data Availability

The data that support the findings of this study are available from the corresponding author upon reasonable request.
